# Development of a Standardized and Safe Airborne Antibacterial Assay, and Its Evaluation on Antibacterial Biomimetic Model Surfaces

**DOI:** 10.1371/journal.pone.0111357

**Published:** 2014-10-31

**Authors:** Ali Al-Ahmad, Peng Zou, Diana Lorena Guevara Solarte, Elmar Hellwig, Thorsten Steinberg, Karen Lienkamp

**Affiliations:** 1 Department of Operative Dentistry and Periodontology, Center for Dental Medicine, Albert-Ludwigs-Universität, Freiburg, Germany; 2 Freiburg Institute for Advanced Studies (FRIAS), Albert-Ludwigs-Universität, Freiburg, Germany; 3 Department of Microsystems Engineering (IMTEK), Albert-Ludwigs-Universität, Freiburg, Germany; 4 Oral Biotechnology, University Medical Center of the Albert-Ludwigs-Universität, Freiburg, Germany; National Central University, Taiwan

## Abstract

Bacterial infection of biomaterials is a major concern in medicine, and different kinds of antimicrobial biomaterial have been developed to deal with this problem. To test the antimicrobial performance of these biomaterials, the airborne bacterial assay is used, which involves the formation of biohazardous bacterial aerosols. We here describe a new experimental set-up which allows safe handling of such pathogenic aerosols, and standardizes critical parameters of this otherwise intractable and strongly user-dependent assay. With this new method, reproducible, thorough antimicrobial data (number of colony forming units and live-dead-stain) was obtained. Poly(oxonorbornene)-based Synthetic Mimics of Antimicrobial Peptides (SMAMPs) were used as antimicrobial test samples. The assay was able to differentiate even between subtle sample differences, such as different sample thicknesses. With this new set-up, the airborne bacterial assay was thus established as a useful, reliable, and realistic experimental method to simulate the contamination of biomaterials with bacteria, for example in an intraoperative setting.

## Introduction

Antimicrobial in-vitro testing is crucial for the design, development and in-vivo performance prediction of biomaterials. However, to get meaningful data, it is important to perform these antimicrobial assays with clinically relevant bacterial strains, and to simulate realistic infection scenarios. In surgery, the sources of contamination found within operating theaters are diverse. They comprise, among others, microbial transport through moving medical personnel, locally formed aerosols (including sneezing/coughing), or building-related sources such as ventilation and air condition [1]–[4]. All in all, ‘Sterile implant surgery may be considered a myth’ [2]. In this context, the airborne infection pathway is of particular relevance, and thus there is a need for an airborne antimicrobial assay that simulates non-contact microbial infection.

There are only a few papers in the literature that actually use airborne antimicrobial assays. In the examples known to us, a commercial chromatography sprayer [5]–[7] was used to produce an aerosol inside a laminar flow box. In short, the sprayer was filled with a suspension of bacteria, and an aerosol was formed by pressing a hand-held balloon. This method is problematic from multiple points of view:

- From the health and safety perspective, creating infectious microbial aerosols in a non-enclosed work space is a problem, because this can distribute pathogens through the lab and jeopardize the safety of laboratory staff. It is therefore legally prohibited within the European Union. The aerosols could also contaminate the laboratory-specific culture collection and thereby ruin the work basis of an entire lab. Obviously, the method is particularly problematic for human pathogenic and/or antibiotic resistant bacteria. Since many clinically relevant strains are highly pathogenic and multi-drug resistant, but have to be tested during biomaterials development, the need for a safe way to perform this assay is evident.
- From a methodological point of view, the above described airborne assay is problematic because it is extremely user-dependent, and it is difficult to generate reproducible results. Parameters such as the bacteria density in the aerosol obtained from an unstirred bacterial suspension, the space between the hand-held sprayer and the target material, the spraying angle, and the spraying pressure can be so far only poorly controlled. The number of colony forming units per spray event thus varies tremendously, which makes it difficult to quantify the data obtained and extract meaningful microbiological results.

**Figure 1:**
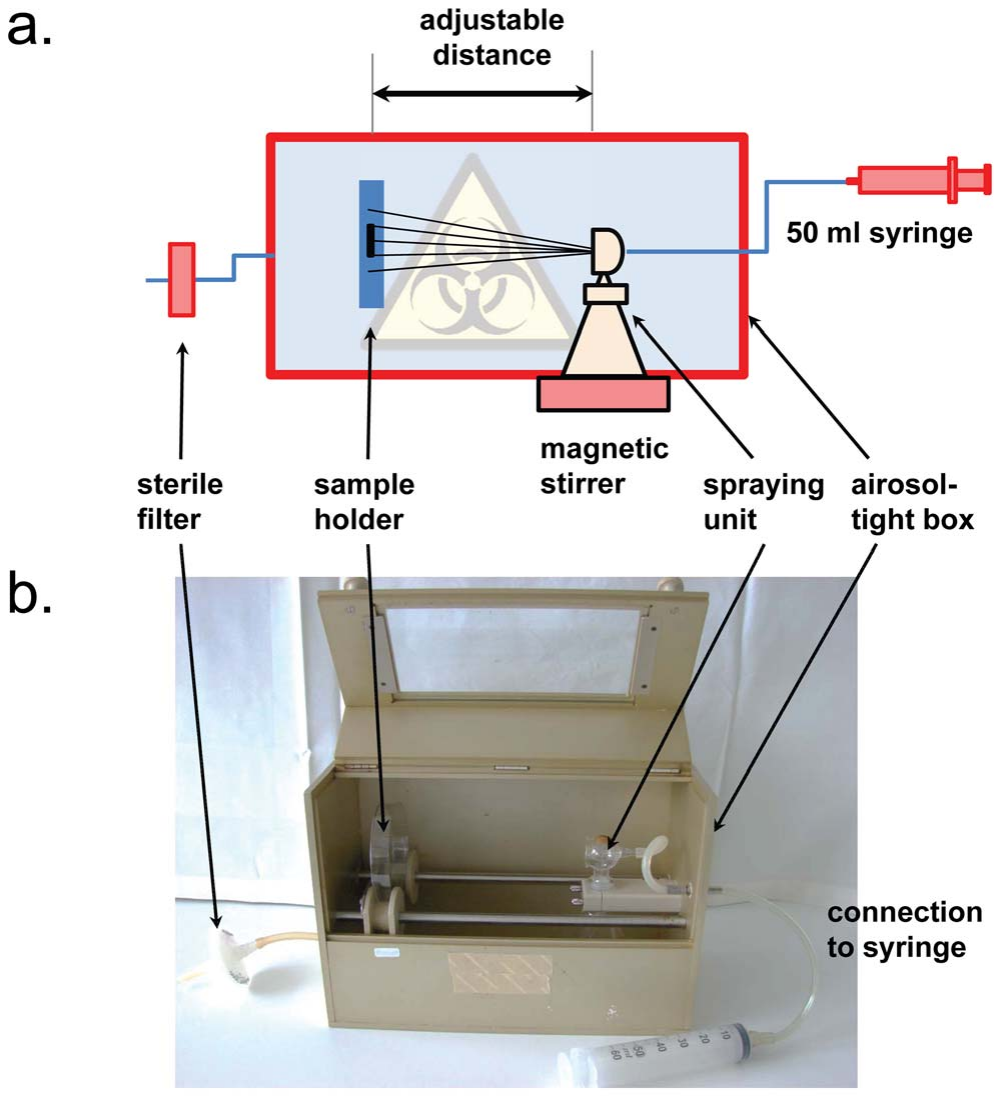
A new experimental set-up for the safe handling of pathogenic bacterial aerosols in the airborne antimicrobial assay is shown. **a)** Schematic diagram of the set-up. The distance between the sprayer and the biomaterial, the spraying angle, the air pressure, and the bacterial concentration in the aerosol can be precisely controlled. **b)** Photograph of the set-up prototype. The construction can be autoclaved as a whole, enabling the safe handling of biohazardous pathogens in a microbiological laboratory.

**Figure 2:**
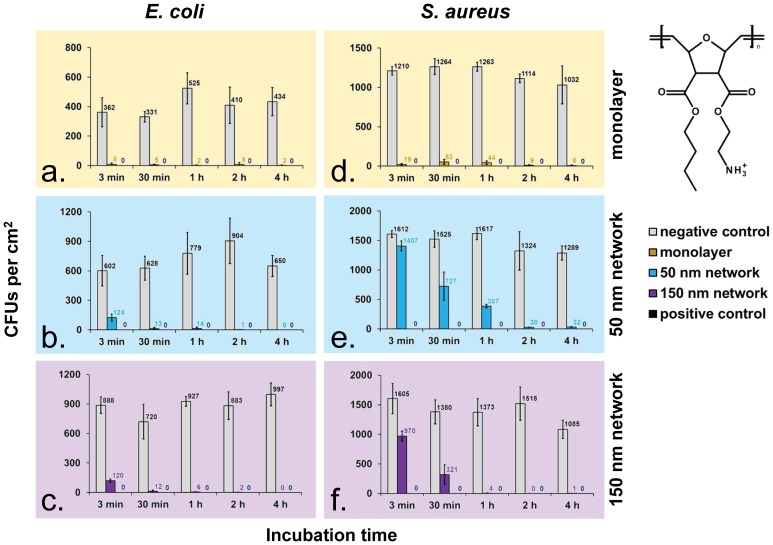
Airborne antimicrobial assay performed with the new set-up. Two different bacteria (a–c: *Escherichia coli*, d–f: *Staphylococcus aureus*) and three different materials (a & d: SMAMP monolayer, b & e: 50 nm thick SMAMP network, c & f: 150 nm thick SMAMP network) were used. The number of colony forming units (CFUs per cm^2^) after various incubation times is given. All values are duplicates with 5 replicates each. The negative control are uncoated silicon wafers. On positive control (silicon wafers immersed in chlorhexidine), no CFUs could be detected (100% killing) in all experiments.

We have therefore developed an airborne assay which allows the reproducible, safe spraying of high-risk pathogenic bacteria on biomaterials. For this purpose, we have constructed a device in which the aerosol is contained during the entire assay, and which can be autoclaved as a whole after the experiment (Fig. 1). Additionally, the bacterial suspension can be stirred, and parameters like bacteria concentration, target-sprayer distance, and spraying conditions can be defined. It turns out that these apparently trivial modifications are crucial for the data quality. We have evaluated this new set-up with antimicrobial polymer coatings, more specifically with poly(oxonorbornene)-based synthetic mimics of antimicrobial peptides (SMAMPs) [8]–[13]. We have selected one particular polymer of this group (Fig. 2), which we previously identified as highly antimicrobially active [8], [12]. Using this compound, we identified and optimized critical set-up parameters such as incubation time, spraying volume, and spraying distance, and standardized the assay. We then tested materials with different layer thicknesses to check if we can see differences even between very similar samples. Our data demonstrates that the results are reproducible and that even subtle trends in the antimicrobial data, such as the difference in activity between a polymer monolayer, a 50 nm thick network and a 150 nm thick network, could be detected. We now have a working method at hand, which will enable the biomaterials community to safely and reproducibly perform meaningful airborne antimicrobial assays even with dangerous microbial aerosols.

## Material and Methods

### Instrument design for the airborne antimicrobial assay

We designed and fabricated a new experimental set-up that allows safe handling of pathogenic aerosols [14]. A diagram of this set-up and a photograph of the prototype are shown in Figure 1. The following requirements had to be fulfilled:

- obtaining a homogenous bacterial suspension with a well-defined optical density and number of colony forming units;
- reproducible spraying of a constant amount of the bacterial suspension;
- multiple specimens of the material should be sprayed in each experiment;
- the bacterial aerosols formed must not enter the laboratory;
- after the experiment, the entire contaminated set-up should be autoclavable without damage to any of its components.

As shown in Fig. 1b, the actual sprayer was enclosed in a box made from aluminum, with a tightly closing lid (aluminum with a build-in glass window). The sample holder was made from autoclavable polyether ether ketone (PEEK) and was mounted on an aluminum rod to adjust the sprayer-sample distance. A commercial chromatography sprayer (VWR, Bruchsal, Germany) containing a magnetic stirring bar was placed opposite the sample holder. The sprayer was connected by a silicone tube to a 50 mL syringe. Using this syringe, a defined volume of air could be pushed through the bacterial suspension to create the bacterial aerosols. To allow pressure compensation without contaminating the outside of the device, the aluminum container was equipped with a sterile autoclavable polytetrafluoroethylene (PTFE) syringe filter (Sartorius, Göttingen, Germany, pore size of 0.2 µm). To keep the bacterial suspension homogenous, it was stirred with a magnetic stirrer during the experiment.

### Fabrication of antimicrobial test surfaces

The antimicrobial surfaces were fabricated as described previously [11]. In short, a solution containing the antimicrobial SMAMP polymer was spin-coated onto a silicon wafer treated with a surface-attached UV-active crosslinker. The polymer was then surface-immobilized using UV-light. This caused covalent cross-linking between the surface and the polymer chains and formed a polymer monolayer on the surface. For the two SMAMP networks, an additional cross-linker was added to the polymer solution prior to spin coating to enable inter-chain cross-linking. The samples were then washed with solvent and immersed into hydrochloric acid to activate the antimicrobial function. Table 1 summarizes the sample fabrication parameters. Polymer synthesis and characterization, surface functionalization and surface coating characterization are described in the supporting information (File S1).

**Table 1:** Polymer sample fabrication parameters: polymer concentration, cross-linker concentration, spin coating conditions and cross-linking conditions for the polymer monolayer and networks; DMPAP  = 2,2-dimethoxy-2-phenylaceto-phenone.

Sample	c (polymer)/mg mL^−1^	c (cross linker)/mg mL^−1^	Solvent/mL	Spin coating conditions	Additives	Cross linking conditions
mono-layer	10	-	CH_2_Cl_2_: Toluene = 1∶4	30 sec, 3000 rpm	-	254 nm, 10 min
50 nm network	9.5	6.2	CH_2_Cl_2_: Toluene = 25∶80	30 sec, 3000 rpm	DMPAP	254 nm, 30 min
150 nm network	15.4	10	CH_2_Cl_2_: Toluene = 25∶40	30 sec, 3000 rpm	DMPAP	254 nm, 30 min

### Airborne antimicrobial activity assay

All bacterial strains used in this study were maintained routinely with weekly sub-culturing on Columbia blood agar (CBA, Oxoid, Wesel, Germany). Long-term storage of these bacteria was at −80°C in basic growth medium containing 15% (v/v) glycerol according to Jones et al. [15] and as described earlier [16].

The basis of the antimicrobial activity assay was the Japanese Industrial Standard JIS Z 2801∶2000, a water-borne test for antibacterial activity and efficacy, as described by Madkour et al. [6]. Importantly, the bacterial inoculation through pipetting a bacterial suspension onto the test material in the JIS assay was replaced by spraying the bacterial suspension on the material in the airborne antimicrobial assay. Additionally, we used our own bacterial strains as described in the following text. All other steps are similar. Two bacterial strains, *Staphylococcus aureus* ATCC 29523 as a Gram-positive example, and *Escherichia coli* ATCC 25922 as a Gram-negative example, were tested. Overnight cultures were prepared in tryptic soy broth (TSB, Merck, Darmstadt, Germany). A log-phase culture was prepared from an overnight-culture by transferring a specific volume into fresh TSB culture medium and incubating for 3–4 h. The optical density of this culture was measured using a Smart-Spec plus spectrophotometer (Bio-Rad, Life Science Group, Hercules, USA) at 595 nm. A bacterial solution with a concentration of ca. 10^6^ colony forming units (CFU) per ml was prepared for each bacterial strain by dilution in 0.9% saline solution. The bacterial solution was transferred into the sterilized sprayer containing a stirring bar. The sprayer was then positioned in the spraying set-up described above at a distance of 15 cm from the sample. The device was placed on a magnetic stirrer, and the bacterial suspension was sprayed using compressed air from a 50 ml syringe as shown in Fig. 1. Pumping a fixed volume of air to drive the sprayer was operated manually.

The test samples were then transferred into a sterile petri dish and incubated for 5 min, 30 min, 1 h, 2 h, and 4 h in a humid chamber at 37°C under aerobic conditions and 5% CO_2_ (capnophilic conditions). For each incubation period, five test samples, five positive controls which were previously immersed in the antiseptic chlorhexidine (Fagron, Barsbüttel, Germany), and five negative controls (uncoated samples), were tested. After incubation, 50 µl of sterile 0.9% saline solution was added with a pipette onto the sprayed area of the tested surface, and left for 2 minutes. To ensure removal of the bacteria from the surface, the solution was pumped back and re-pipetted twice, then pumped back a third time and spread on a Columbia blood agar plate (CBA). The agar plates were incubated for two days at 37°C with 5% CO_2_. The colony forming units (CFUs) were counted using the Gel Doc EQ Universal Hood (Bio-Rad Life Science Group, Hercules, USA). The killing effects of the coated surface were measured by comparing the CFU number with the results from the uncoated wafers (negative control) and with the wafers which were previously immersed in 0.2% chlorhexidine digluconate (positive control). The percent killing was calculated by the following method: % Survival  =  [(test sample CFU – positive control CFU)/(negative control CFU – positive control CFU)] ×100.

### Airborne antimicrobial assay with Live/Dead staining

To differentiate between membrane-compromised (“dead”) bacteria, and bacteria with intact membrane (“live”) directly on the surface, SYTO 9 stain and propidium iodide (PI) (Live/Dead BacLight Bacterial Viability Kit, Life Technologies GmbH, Darmstadt, Germany) were used as described earlier in detail [17], [18]. The fluorescent agent was dissolved in a 0.9% saline (NaCl) solution to a final concentration of 0.1 nmol ml^−1^, each. The bacterial culture (10^8^ colony forming units per ml) to be sprayed was prepared in this live/dead solution and immediately sprayed on the different samples. As previously, one wafer which had been immersed in chlorhexidine (positive control), and one untreated wafer (negative control), was tested with the polymer-coated samples. All samples were then left in a dark chamber for 10 min at room temperature. The samples were covered with a cover slip (Langenbrinck, Emmendingen, Germany) and inserted into the wells of multi-well plates (12-well plate; Greiner bio-one, Frickenhausen, Germany). These were kept in a dark chamber until the actual fluorescence analysis. Microscopic analysis was conducted using the Keyence BZ-9000 fluorescence microscope (Keyence Germany, Neu-Isenburg, Germany) to visualize and quantify “dead” (red) and “live” (green) bacteria.

## Results

**Figure 3:**
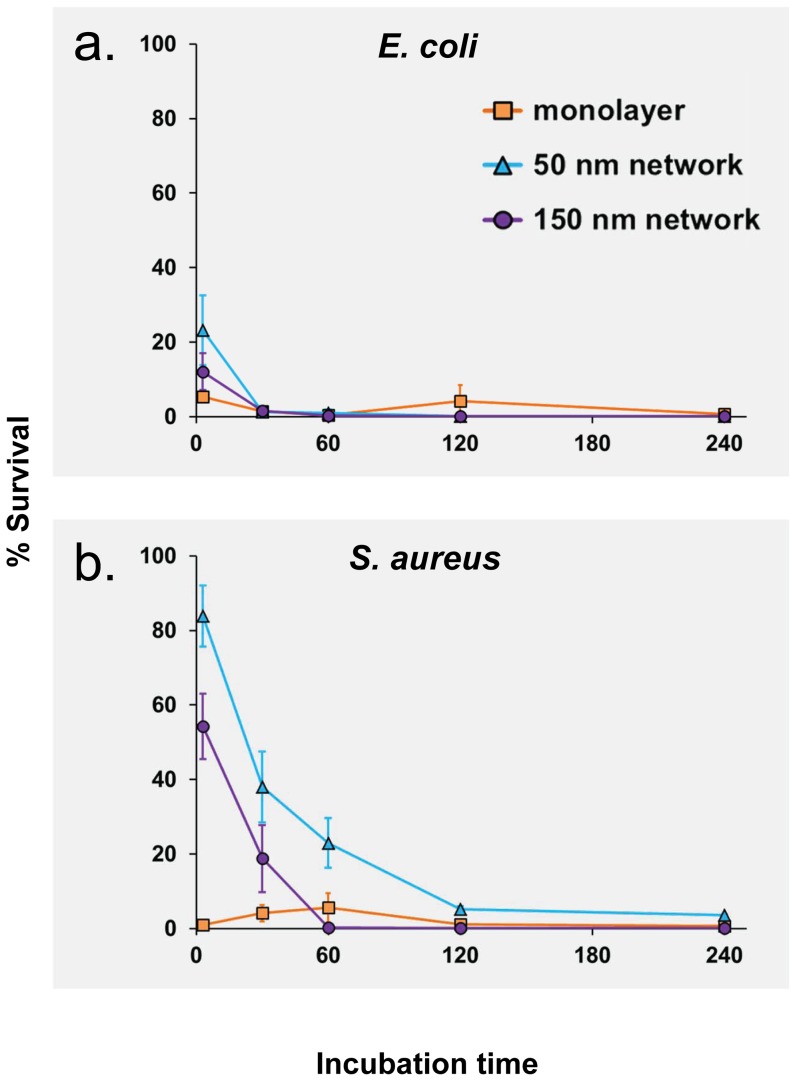
The effect of incubation time on the number of colony forming units for *Escherichia coli* (a) and *Staphylococcus aureus* (b). The percentage of bacterial survival was calculated relative to the negative control, which was defined as 100% survival for each sample set. All values are duplicates with 5 replicates each.

Three types of antimicrobial polymer surfaces were tested using the above described airborne antimicrobial assay: a polymer monolayer, a 50 nm thick polymer network, and a 150 nm thick polymer network. The results are shown in Fig. 2. For each data point, the assay was performed in duplicate, with five samples per repetition. Two bacteria (*E. coli* and *S. aureus*), and five different time points (3 min, 30 min, 1 h, 2 h, and 4 h) were tested. Chlorhexidine sprayed onto uncoated silicon wafers was used as a positive control, and uncoated silicon wafers were used as a negative control. Fig. 2a–c show the absolute number of colony forming units for *E. coli*, and Fig. 3a shows percent survival of adherent *E. coli* bacteria. The data shows (in the negative control) that consistent and reproducible amounts of colony forming units (CFUs) were obtained for all samples. The error bars were acceptable not veil the differences or trends between samples.

**Figure 4:**
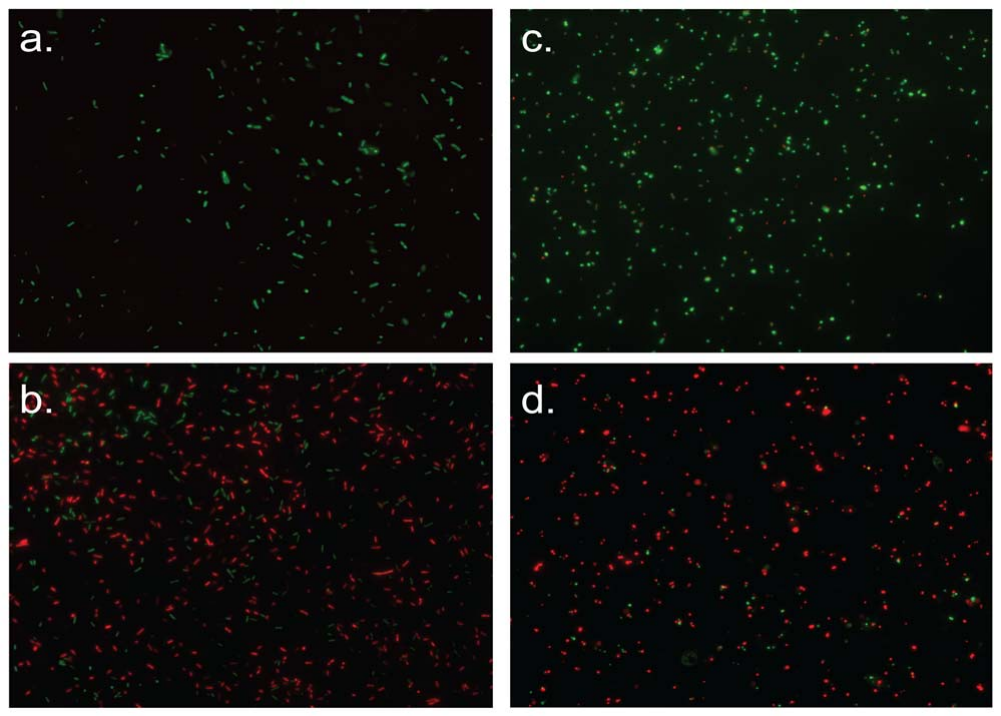
Fluorescence microscopy images of the adherent bacterial cells after the use of live/dead staining. Each image is an overlay of the green and red fluorescent channels. Green spots represent active, membrane-intact bacteria, red spots are “dead”, membrane compromised bacterial cells. The incubation time was 2 h. a) *Escherichia coli* (negative control, approximately 100% “live” cells), b) *Escherichia coli* (SMAMPs-coated wafers, 75% “dead” cells), c) *Staphylococcus aureus* (negative control, 90% “live” a cells), d) *Staphylococcus aureus* (SMAMPs-coated wafers, 86% dead cells).

For *E. coli*, the means of CFUs in the negative control ranged from 331–997 CFU cm^−2^, with mean values of 331–525 CFU cm^−2^ for the SMAMP monolayer, 602–904 CFU cm^−2^ for the 50 nm SMAMPs network, and 720–997 CFU cm^−2^ for the 150 nm SMAMPs network. The mean CFU number of the negative control for each sample set was fixed as 100%, and all other values were calculated in relation to this value. The positive control (silicon wafers immersed into chlorhexidine solution) killed 100% of the adherent bacteria in all cases. As shown in Fig. 3a, the polymer coated samples each killed the adherent *E. coli* bacteria to a high level of at least 80% after only 3 min. After ≥30 min, the bactericidal effects were roughly comparable with chlorhexidine (98–100% killing of the adherent bacteria). As shown in Fig. 2d–f and Fig. 3b, the interaction of the polymer-coated materials with *S. aureus* was much more time-dependent. The 50 nm and 150 nm thick SMAMPs-networks needed significantly longer to obtain near-quantitative killing of *S. aureus*. Surprisingly, the SMAMP monolayer killed 99% *S. aureus* after only 3 min, while the 50 nm and 150 nm networks only killed 17% and 63%, respectively. The 50 nm network eliminated up to 97% *S. aureus* after 4 h, and the killing rate of the 150 nm network reached 100% after 60 min. The live dead staining technique confirmed the high antimicrobial effects of SMAMPs-coated surfaces for both *E. coli* and *S. aureus*. This is shown for the 150 nm network in Fig. 4. On the negative controls (Fig. 4a for *E. coli* and Fig. 4c for *S. aureus*), mainly green (membrane-intact, “live”) bacteria were detected, whereas the SMAMPs-coated surfaces had a much higher number of red (membrane-compromised, “dead”) bacteria. Specifically, up to 76% of adhered *E. coli* cells were killed on SMAMPs coated wafers (Fig. 4b), compared to ∼100% survival of *E. coli* on the control (Fig. 4a). Considering the 10 min incubation time used in this experiment, this is perfectly in line with the above reported CFU results (Fig. 3a). For *S. aureus,* the killing rate on the polymer coated wafer surface was 86% for *S. aureus* (Fig. 4d), with 90% survival of *S. aureus* on the negative control (Fig. 4c). This is significantly more “killing” than in the CFU data for *S. aureus* (Fig. 3b) and indicates that some of these bacteria are membrane-compromised, but not dead. The fact that the negative control has only 90% survival supports this interpretation. Importantly, however, the images in Fig. 4 all show a homogenous distribution of the bacterial cells on all surfaces, with no cluster formation and no debris. This indicates that our spraying assay indeed yields well-defined, thorough results.

## Discussion

Biomaterial-associated infections are diverse and a major concern in different medical and surgical fields. For example, contact lenses are predominantly infected by *Pseudomonas aeruginosa* [19], whereas prosthetic joint infections are typically due to staphylococci [20]. Some particularly feared resistant bacteria such as Methicillin-resistant *Staphylococcus aureus* (MRSA) strains are meanwhile established pathogens in hospitals and the community [21]. Infections caused by multi-resistant extended spectrum beta-lactamases (ESBL)-producing bacteria (*Escherichia coli* and *Klebsiella pneumoniae*) have also been increasingly reported in orthopedic and trauma surgery [22]. One important origin of these infections is the contamination of the biomaterial surfaces by microorganisms during handling in the operating room. Such contaminations may turn into persistent biofilm infections, which are difficult to treat with antibiotics. They can cause life threatening infections in patients, as well as high additional costs [2]. This is why the development of antimicrobial surfaces for medical and surgical devices is an important field. Independently of the strategy used for preparing such materials, the resulting surfaces must then be tested *in vitro* for their antimicrobial activity. For this, a broad panel of potential pathogens needs to be studied in realistic experimental settings. Using a waterborne assay such as the above mentioned Japanese Industrial Standard Z 2801∶2000 for testing the antimicrobial properties of biomaterials is useful, especially for testing biofilm formation. However, the contamination of a surface by waterborne pathogens does not reflect the reality of the initial contamination of biomaterials or health care devices in operating rooms and hospitals.

So far, only a few studies have tried to simulate the microbial contamination of biomaterial surfaces using an airborne assay [5]–[7], and all of them included the uncontrolled production of pathogenic aerosols. These aerosols have to be avoided in microbiological laboratories for work safety and legal reasons (European Directives 90/219/EEC). We therefore developed the here presented new set-up for the safe and reproducible spraying of potentially human-pathogenic and multi-resistant bacteria on material surfaces, without exposing the laboratory staff or the lab to microbial aerosols. We tested and optimized this apparatus for the airborne assay on antimicrobial polymer surfaces made from synthetic mimics of antimicrobial peptides (SMAMPs). Besides taking care of safety considerations, our method standardizes several parameters that are critical for the airborne assay:

- the distance between the sample holder and the sprayer, and the spraying angle,
- the concentration of the bacterial suspension from which the aerosol is formed (due to stirring), and the air pressure pulse that forms the aerosol.

As a result, a standardized and reproducible amount of bacterial aerosols could be generated, as revealed by the low standard deviation values. It was already pointed out by Haldar et al. [5] in their protocol for the airborne assay that the spraying step is critical when this testing is conducted. This means that all parameters involved in this step need to be precisely controlled, and special care must be taken that the bacterial suspension is uniform. Since bacteria can also form chains and small flocks in planktonic cultures, resulting in high variation in the determined colony forming unit (CFU) number, we believe that stirring the solution during the spraying event is crucial. When using a low range of only a few hundred CFUs on a surface of 1 cm^2^, which simulates the wound surface contamination during surgery [23], a homogeneous bacteria distribution in the suspension is particularly important.

High antimicrobial effects in the bactericidal range of 99.9% could be shown for some of our SMAMPs-coated materials, even after a short incubation time. The antimicrobial activity revealed by determination of the colony forming units (CFU) was confirmed by a live/dead staining technique. As agitating or treatment in an ultrasonic bath may not desorb all adherent bacteria in the airborne or waterborne assay that need to be plated out, the live/dead stain is useful because it gives an unbiased in-situ representation of the sample surface. Such critical combination of methods was previously shown to be helpful in quantifying the initial adhesion on dental surfaces *in situ* without disturbing natural adhesion [24]. The live/dead staining technique proved that the spraying process on the surface, considered to be a critical point for an airborne assay [5], delivered a homogenous distribution of bacterial cells, demonstrating the appropriateness of the device used in our present study, and the uniform activity of the surfaces tested.

The effect of increased bacterial killing with increased contact time was expected and matches the model of Busscher and van der Mei [25] who described a lethal adhesion force regime as being a response of bacteria to a surface. This regime was supported by the observations of Liu et al. [26] who correlated strong adhesion forces with stress deactivation of cell membranes and to an increase of dead adherent bacteria. Such strong adhesion forces can occur between bacteria which are mostly negatively charged, and positively charged surfaces, such as quaternary ammonium-coated surfaces that were reported to kill adherent bacteria [27]. Similar lethal strong adhesion forces can be hypothesized for the interaction between the positively charged SMAMPs surfaces and the bacteria tested in this study. We and others have previously reported that SMAMPs in solution were membrane active [12], [28]. Due to this unspecific mechanism of action, in contrast to the targeted action of antibiotics, a much lower probability of resistance formation can be expected from bacteria exposed to SMAMPs.

In summary, we have presented a safe and reliable method to simulate real-life infection scenarios with aerosols of pathogenic bacteria. We have tested the method on model surfaces coated with polymer-based synthetic mimics of antimicrobial peptides (SMAMPs), and showed that these SMAMPs are highly antimicrobially active, in some cases even bactericidal. With this assay and experimental set-up, we are offering the biomaterials community a useful tool for antimicrobial testing. Future work will be dedicated to optimize this prototype, for example by automated aerosol pulse generation. This would reduce the possibility of errors caused by manual operation in different laboratories.

## Supporting Information

File S1 **Polymer synthesis and characterization, surface functionalization and surface coating characterization are described in the supporting information.**
(PDF)
