# Supplementary material for: Development of a Standardized and Safe Airborne Antibacterial Assay, and Its Evaluation on Antibacterial Biomimetic Model Surfaces
Source: PLoS One. 2014 Oct 31;9(10):e111357. doi: 10.1371/journal.pone.0111357 (PMC4216082; doi:10.1371/journal.pone.0111357)
Supplement: File S1 — Polymer synthesis and characterization, surface functionalization and surface coating characterization are described in the supporting information. (PDF) [file pone.0111357.s001.pdf]

Development of a standardized and safe airborne antibacterial assay, and its evaluation on antibacterial biomimetic model surfaces – Supporting information

Ali Al-Ahmad[1*,#], Peng Zou[2,3], Diana Lorena Guevara Solarte,[1,3] Elmar Hellwig[1], Thorsten Steinberg[4], Karen Lienkamp[2,3,#]

[1] Department of Operative Dentistry and Periodontology, Center for Dental Medicine, Albert-Ludwigs-Universität, Freiburg, Germany

[2] Freiburg Institute for Advanced Studies (FRIAS), Albert-Ludwigs-Universität, Freiburg, Germany

[3] Department of Microsystems Engineering (IMTEK), Albert-Ludwigs-Universität, Freiburg, Germany

[4] Oral Biotechnology, University Medical Center of the Albert-Ludwigs-Universität, Freiburg, Germany

*Corresponding Author:

Department of Operative Dentistry and Periodontology,

Center for Dental Medicine,

Albert-Ludwigs-Universität, Freiburg, Germany,

Hugstetter Straße 55,

D-79106 Freiburg, Germany

Phone: +49 761 27048940, Fax: +49 761 27047620

E-Mail: ali.al-ahmad@uniklinik-freiburg.de

[#]These authors contributed equally to this work.

Synthesis of SMAMP Precursor.

Monomer and SMAMP precursor were synthesized as described in literature.[1][2] Monomer (1.0 g, 2.6 mmol) and Grubbs' 3rd generation catalyst (2.9 mg, 0.002 mmol) were dissolved respectively in DCM (4 mL and 1 mL). Both solutions were stirred at room temperature for 30 min. The monomer solution was then added to the catalyst solution in one shot and the reaction mixture was stirred for another 30 min at room temperature. After adding ethylvinyl ether (2.0 mL, 1.5 g, 20 mmol) and stirring for 1 h at room temperature, the solution was concentrated by evaporating the solvent under reduced pressure. The SMAMP precursor was then precipitated into n-hexane (1 L) and dried under dynamic high vacuum overnight. The polymer was characterized by ^{1}H-NMR (see below) and gel permeation chromatography (see below). The NMR signals of the polymer matched those in the literature and the GPC analysis (PSS SDV column, Chloroform, 30°C, 1 mL·min^{-1}) indicated a molecular weight of 180 000 g·mol^{-1} with a polydispersity of 1.1. Characterization data of the SMAMP precursor is also given in Fig. S1 a-c.

Fig. S1. Physical characterization of the SMAMP precursor polymer. a) Chemical structure and NMR peak assignment; b) NMR spectrum; c) GPC elugram.

a)

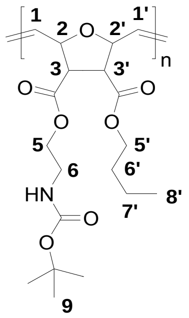

^{1}H-NMR of SMAMP precursor (250 MHz, $CDCl_3$): 0.94 (t, 8'-CH_3), 1.28–1.51 (m, 7'-CH_2 & 3 × 9-CH_3), 1.62 (m, 6'-CH_2), 3.13 (br m, 3-H & 3'-H), 3.37 (br m, 6-CH_2), 4.01-4.24 (m, 5-CH_2 & 5'-CH_2), 4.73 (br m, 2-H & 2'-H, trans), 5.13 (br m, 2-H & 2'-H, cis), 5.41 (br, NH), 5.61 (br m, 1-H & 1'-H, cis), 5.91 (br m, 1-H & 1'-H, trans).

b)

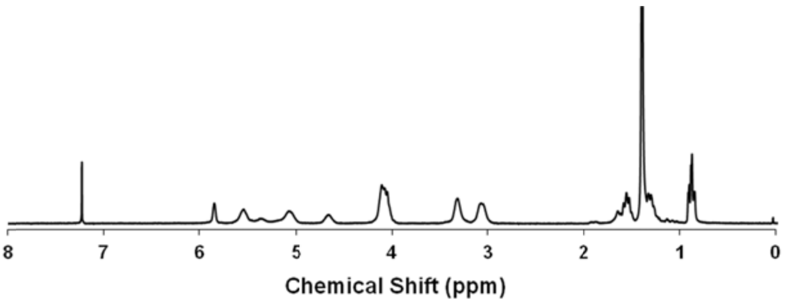

c)

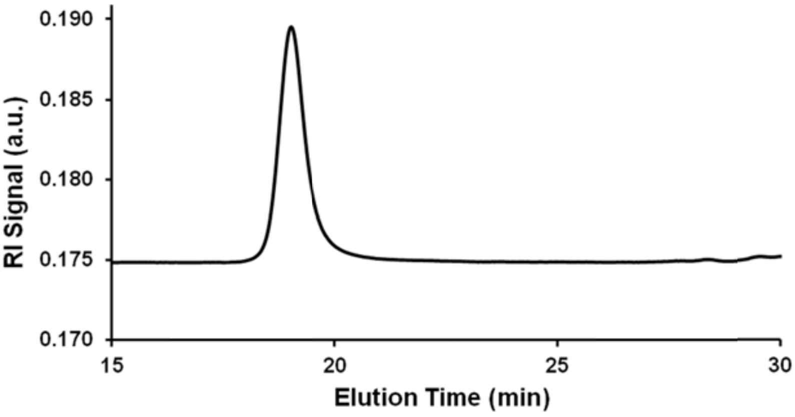

Functionalization of the silicon wafers with 3EBP-silane.

A one-side-polished standard Si (CZ) wafer (525 ± 25 μm thick, orientation [100]) was washed with toluene, DCM, acetone and isopropyl alcohol. A solution of 3EBP-silane (20 mg·mL^{-1} in toluene), which was synthesized as described in the literature[3], was then spin coated (1000 rpm, 120 sec) on the wafer and immobilized by curing on a hot plate for 30 min at 100°C. The unreacted 3EBP-silane was washed with toluene, DCM, acetone and isopropyl alcohol. The wafer was then dried under a nitrogen flow. In the end, the functionalized silicon wafer was cut into small pieces (1.5 ×1.5 cm^2). The functionalization process is illustrated in Fig. S2.

Fig. S2. Functionalization of silicon wafers with 3EBP-silane

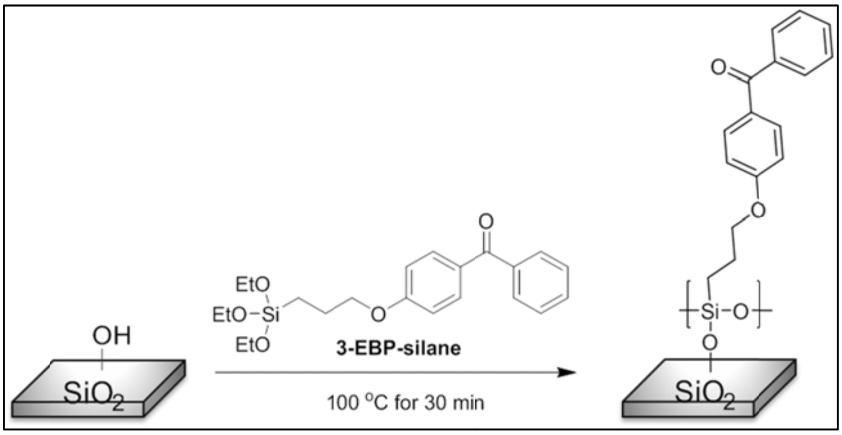

Immobilization of the SMAMP precursor on the functionalized silicon wafer.

1. *Immobilization of SMAMP precursor monolayer on functionalized silicon wafer.*

A solution of the SMAMP precursor (10 mg) in a mixture of DCM and toluene (1:4, 1 mL) was spin coated (3000 rpm, 30 sec) on the 3-EBP treated silicon wafer. The polymer was immobilized on the wafer surface by irradiation at 254 nm for 10 min in a BIO-LINK Box (Vilber Lourmat GmbH). The unreacted polymer was then washed with DCM for three times and the sample was dried under N_2-flow.

2. *Immobilization of SMAMP precursor network on functionalized silicon wafer.*

A stock solution (Solution A) was prepared by dissolving pentaerythritol-tetrakis-(3-mercaptopropionate) (1 mL, 1.3 g, 2.6 mmol) and 2,2-dimethoxy-2-phenylaceto-phenone (4 mg, 0.02 mmol) in DCM (50 mL). After dissolving the SMAMP precursor (10 mg, 0.026 mmol) in Solution A (0.25 mL), different amounts of toluene (0.4 mL and 0.8 mL) was added in order to obtain different SMAMP network thicknesses (around 150 nm and 50 nm). The polymer solution was then spin coated (3000 rpm, 30 sec) on the 3-EBP treated silicon wafer pieces (1.5 ×1.5 cm^2). The SMAMP precursor network was formed and immobilized on the wafer surface by cross-linking at 254 nm for 30 min in a BIO-LINK Box (Vilber Lourmat GmbH). In the end, the unreacted polymer chains were removed by immersing the network in DCM for 4 h. The immobilization process is illustrated in Fig. S3.

Fig. S3: Immobilization of the SMAMP precursor network on the functionalized silicon wafer

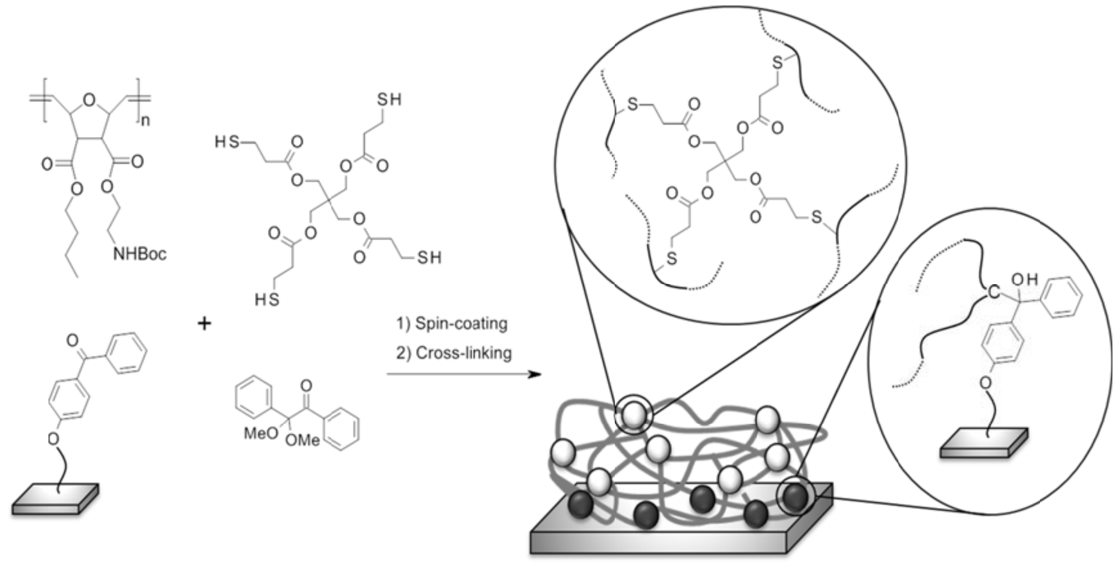

SMAMP activation.

The wafer covered with SMAMP precursor monolayer or network was immersed in HCl (4 M in dioxane) for 12 hours to obtain the complete deprotection of the Boc-protected amine groups. Before antimicrobial test, the sample was washed for three times with ethanol and dried under N_2-flow.

Surface characterization.

Fourier transform infrared spectroscopy (*FTIR).* Double side polished silicon wafers were used as substrates for the FTIR experiments. FTIR was performed with the SMAMP precursor and SMAMP networks with a thickness of around 150 nm, gave sufficient adsorption intensity. The polymer layer was immobilized on one side of a double side polished silicon wafer. The spectra were recorded from 4000 to 400 cm^{-1} with a Bio-Rad Excalibur spectrometer (Bio-Rad, München, Germany), using a spectrum of the blank double side polished silicon wafer as background. The FTIR spectrum of the SMAMP network before and after deprotection is given in Fig. S4.

Fig. S4. FTIR spectrum of the SMAMP network before and after deprotection.

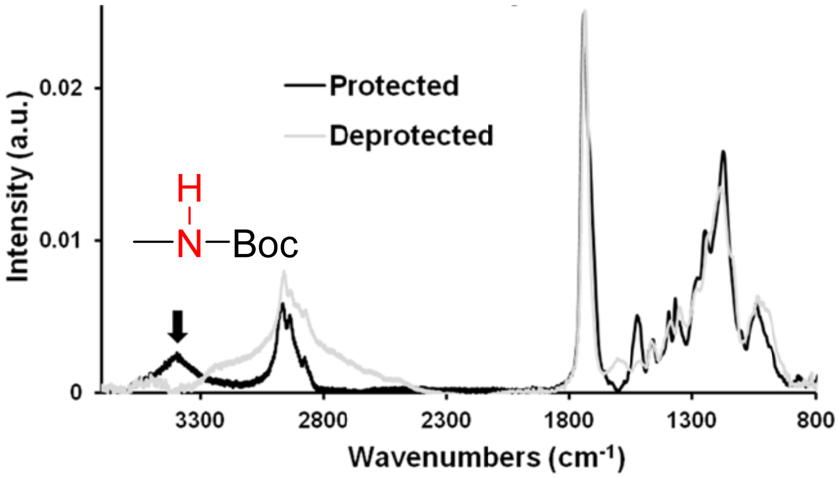

Ellipsometry. The thicknesses of the immobilized polymer layers were measured with the ellipsometer Nanofilm EP3 (Nanofilm Technologie GmbH, Göttingen, Germany). For every sample, three positions on one sample were measured to obtain the average value. A refractive index of 1.5 was assumed for all polymer layers. The ellipsometry data is given in Table S1.

Table S1: Ellipsometry data for the protected and deprotected SMAMP layers.

	**Layer thickness in nm (protected SMAMP)**	**Layer thickness in nm (deprotected SMAMP)**
**Monolayer**	4 ± *1*	3 ± *1*
**Network 1**	62 ± *2*	55 ± *3*
**Network 2**	*178* ± *3*	*153* ± *5*

Contact angle. The contact angle system OCA 20 (Dataphysics GmbH, Filderstadt, Germany) was used to measure the static, advancing and receding contact angles of different sample surfaces. The average value of the contact angle was obtained from four measurements on different positions of one sample. The static contact angles were calculated with the Laplace-Young method, while the advancing and receding contact angles were calculated with elliptical and tangent methods. All contact angle data is given in Table S2.

Table S2: Contact angle data for the SMAMP layers.

	**Static / °**	**Advancing / °**	**Receding / °**
**Monolayer**	75 ± *2*	78 ± *2*	26 ± *2*
**Network 1**	69 ± 3	70 ± *2*	15 ± *3*
**Network 2**	70 ± 3	68 ± 3	17 ± 2

References.

1. Lienkamp K, Madkour AE, Musante A, Nelson CF, Nusslein K, et al. (2008) Antimicrobial Polymers Prepared by ROMP with Unprecedented Selectivity: A Molecular Construction Kit Approach. J Am Chem Soc 130: 9836-9843.
2. Lienkamp K, Madkour AE, Kumar K-N, Nuesslein K, Tew GN. (2009) Antimicrobial Polymers Prepared by Ring-Opening Metathesis Polymerization: Manipulating Antimicrobial Properties by Organic Counterion and Charge Density Variation. Chem Eur J 15: 11715-11722, S11715/11711-S11715/11713.
3. Prucker O, Naumann CA, Rühe J, Knoll W, Frank CW. (1999) Photochemical Attachment of Polymer Films to Solid Surfaces via Monolayers of Benzophenone Derivatives. J Am Chem Soc 121 (38): 8766-8770.
